# Percutaneous Trigeminal Ganglion Balloon Compression Rhizotomy: Experience in 27 Patients

**DOI:** 10.1100/2012/328936

**Published:** 2012-04-01

**Authors:** Tadej Strojnik, Tomaž Ŝmigoc

**Affiliations:** ^1^Department of Neurosurgery, University Clinical Centre Maribor, Ljubljanska 5, SI-2000 Maribor, Slovenia; ^2^Department of Neurosurgery, Faculty of Medicine, University of Maribor, SI-2000 Maribor, Slovenia

## Abstract

*Purpose*. Percutaneous ganglion balloon compression (PBC) is a minimally invasive procedure for treatment of trigeminal neuralgia. *Materials and Methods*. Twenty-seven (19 female and 8 male) patients, who presented with classical symptoms of trigeminal neuralgia, were included. Age ranged from 34 to 91 years (median 62 years), 33 procedures were performed. Duration of the symptoms ranged from 1 year to 30 years (median 5 years). *Results*. After the procedure, pain relief was reported in 25 (93%) patients. In two patients, the pain remained the same. The pain free period ranged from 2 to 74 months (median 15 months). A mean duration of analgesia was longer in patients with ideal pear shape of balloon at the time of the procedure compared to nonideal shape (*P* = 0.01). No major complications occurred in our group of patients. *Conclusions*. Percutaneous trigeminal ganglion balloon compression is a safe, simple, and effective method for temporary pain relief in a selective group of trigeminal neuralgia patients.

## 1. Introduction

The diagnosis and medical management of trigeminal neuralgia (TN) can be one of the most frustrating and rewarding challenges in neurosurgical practice. Exact cause of it is not known, and various types of treatment modalities are possible, and yet no surgical treatment for TN meets ideal requirements [1]. TN is described as sudden, usually unilateral, severe, brief, stabbing recurrent episodes of pain within the distribution of one or more branches of trigeminal nerve, which has a profound effect on quality of life [2, 3]. Patients pain severity correlate with reduced daily functioning and poor health status [4, 5]. All that prompting the search for new operation techniques and the shearing of experiences with existing ones, among them percutaneous balloon compression (PBC) is promising.

According to the classification of the International Headache Society, TN can be classified as classical or idiopathic and on symptomatic or secondary [3, 6]. Burchiel presented diagnostic classification which is driven by the patient's history, where type 1 TN is predominantly episodic and sharp and type 2 TN is constant, dull, and burning in nature [7, 8].

Pathophysiology is not completely understood. Ephaptic conduction caused by segmental demyelination and artificial synapse formation is speculated to be the cause [9–11]. In ephaptic transmission signal from large-diameter partially demyelinated A fibers conduct to small-diameter poorly myelinated A-delta and unmyelinated nociceptive C fibers, which results in paroxysmal facial pain. In pathogenesis of TN neurovascular conflict is seen—vascular compression of the trigeminal nerve at the root entry zone that is most frequently caused by superior cerebellar artery. Other possible causes of demyelination may be tumor of posterior cranial fossa, plaque within brainstem in multiple sclerosis (MS), basilar impression, aneurysm, arteriovenous (AV) malformation, atherosclerosis, and natural aging [9, 11].

Diagnosis of TN is based on history. Classical symptoms of TN include paroxysmal, “electric” pain in the trigeminal distribution on one side of the face; trigger areas that, when stimulated by cold air, washing, speaking, eating, or touch, bring this classic type of pain; periods of remission and exacerbation; pain that is typically more severe in the morning and absent during sleep; periodic pain relief when treated with an adequate trial of carbamazepine. The physical exam is generally normal in TN. Trigeminal sensory deficits, bilateral involvement, abnormal trigeminal reflexes, and herpetic vesicles are signs of symptomatic TN. Magnetic resonance imaging (MRI) is used not only to identify patients with structural causes—tumors, MS, but also to demonstrate neurovascular conflict [6, 9]. In differential diagnostic of TN herpes zoster, dental disease, orbital disease, giant cell arteritis, and intracranial tumor are considered. In one study there were reported six (15.4%) cases of unnecessary dental procedures by patient with TN [3]. The American Academy of Neurology (AAN) and the European Federation of Neurological Societies (EFNS) Guidelines on TN treatment from 2008 recommend medical treatment with carbamazepine and oxcarbazepine or lamotrigine and baclofen as first option [6]. When patients are refractory to medical treatment or side effects of medications exceed patients' tolerability, early surgical therapy is recommended. Various modalities of surgical treatment are possible, from major intracranial operation to minimally invasive percutaneous techniques. Because of the longest duration of pain freedom, as a first surgical option microvascular decompression (MVD) is recommended. But it presents major intracranial operation, which may not be suitable for older, debilitated patients. MVD is also presented with risk of major neurological complications and mortality. Due to their minimally invasive nature and possibility to repeat it are percutaneous procedures widely used methods for the surgical treatment of TN, mostly in cases not suitable for MVD, when patients are not willing to have MVD or they are refractory to previous surgical treatment. One of them is percutaneous ganglion balloon compression (PBC). It was first introduced by Mullan and Lichtor in 1983 [12]. Since then it represent effective, low-cost, simple therapeutic modality for treatment of TN. Compression by PBC selectively injures the myelin present in large myelinated fibers that mediate light touch. PBC is selective for large fibers but not selective for pain originating from a particular trigeminal division [13]. Compression selectively preserves the small unmyelinated fibers that mediate pain and temperature sensation. It does not injure the axons themselves. Balloon compression reduces the sensory neuronal input, thus turning off the trigger to the neuropathic trigeminal pain. The Department of Neurosurgery of University Clinical Center (UCC) Maribor is the first and only center in Slovenia that has used PBC for treatment of TN. In this article our experience with PBC is presented.

## 2. Materials and Methods

Within a ten-year period (from 2000 until 2010), 33 PBC were performed in 27 patients at the Department of Neurosurgery of UCC Maribor. There were 19 female and 8 male patients. Age ranged from 34 years to 91 years (median 62 years). Patients included in the study were suffering from classical symptoms of trigeminal neuralgia, 22 cases had nature of type 1 TN and 11 cases of type 2 TN. One patient had bilateral trigeminal neuralgia, 16 patients were affected on the right side and 10 on the left side. All patients were treated with medications prior PBC without sufficient pain relief. The median duration of the symptoms before PBC was 5 years, ranged from one year to 30 years, 5 patients had already undergone surgery, among them three previously had MVD, one alcohol rhizotomy, and one section of the trigeminal sensory root. On 27 patients 33 procedures were performed—three patients had two procedures on the same side and one patient had four procedures, three on the same side and one on the other side.

### 2.1. Short Procedure Description

**Figure 1:**
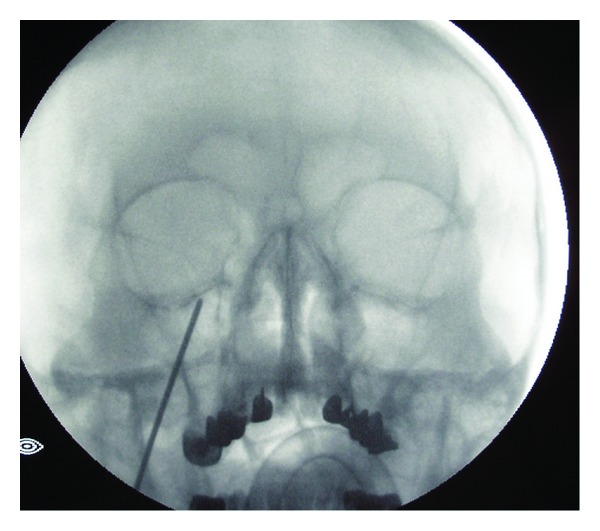
Through the entry point—2.5 cm lateral to the corner of the mouth, a specially designed needle with thin stylet was passed into foramen ovale.

**Figure 2:**
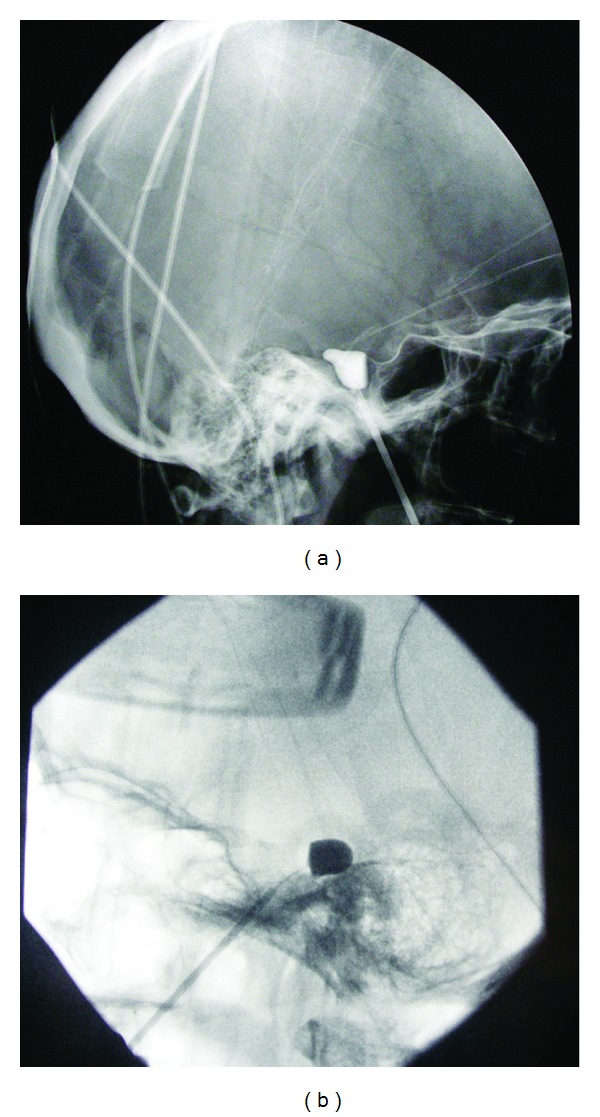
The balloon catheter was introduced through the needle and navigated into Meckel's cave. The shape showed if the balloon was in the appropriate position: (a) ideal pear shape; (b) not ideal shape.

For PBC Mullan percutaneous trigeminal ganglion microcompression set (Cook Vascular Inc., US) was used. Patient was positioned supine on the operating table. Procedures were done in general anesthesia with a short-acting anesthetic agent. X-ray control of pyramid and foramen ovale was performed. A specially designed 14-gauge needle with thin stylet was passed 6 cm into the depth towards foramen ovale through entry point located 2.5 cm lateral to the corner of the mouth (Figure 1). Foramen ovale was entered with dull tip of stylet. X-ray control of needle position was made and the balloon catheter was introduced through the needle and navigated into Meckel's cave. The position of tip was confirmed by using a C-arm fluoroscopic image intensifier in both anteroposterior and lateral views. About 1 mL of water-soluble contrast (iohexol) was injected to inflate the balloon, until ideal pear shape of the balloon was acquired (Figure 2). The duration of inflation depended upon the duration of the disease (longer course of disease, longer time of inflation). After one to five minutes the balloon was deflated and the needle and catheter were removed simultaneously. Firm pressure was applied to the cheek for some minutes. The patients were discharged after an overnight stay.

### 2.2. Patient Database and Statistical Analysis

The medical and surgical records of all patients were retrospectively reviewed and analyzed. The following data were collected: gender, age, side of trigeminal neuralgia, duration of symptoms prior PBC in years, previous surgical procedures, time of balloon inflation in minutes, the shape of the balloon, number of consecutive PBC, type of pain, complications during and after procedure, hypoesthesia after PBC, duration of analgesia after PBC in months, need for analgesics after 12 months, and procedures done after PBC. Descriptive statistical analysis was performed and patients' parameters were statistically analyzed with Fischer exact test for descriptive variables, independent Student's *t*-test for numeric variables and Kaplan-Meier analysis for duration of analgesia. Statistically significant were values with *P* < 0.05. The software used was SPSS 17.0.

## 3. Results

### 3.1. Outcome

**Figure 3:**
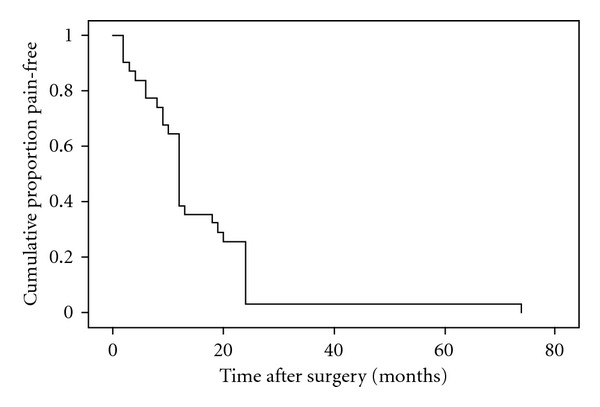
Kaplan-Meier plot illustrating pain-free proportion of patients after a percutaneous trigeminal ganglion balloon compression. Mean pain-free period was 15 months.

After one or more procedures pain relief was reported in 25 (93%) of patients. In two patients the pain remained the same. Pain-free period in remaining 25 patients ranged from 2 to 74 months with mean value 15 ± 13.2 months (Figure 3).

Twelve months after the PBC patients needed less analgesics than before PBC in 22 (67%) cases and no analgesics in one (3%) case. In 8 (24%) cases the needed dose of analgesic was the same and in two (6%) cases patients needed more analgesic than before PBC. In the case of pain recurrence a brief period of increased medical therapy was sufficient to control the pain. In refractory patients another procedure was performed. After repeated procedure patients remained pain-free with minimal medication for additional 6–12 months. There were 22 cases of type 1 TN and 11 cases of type 2 TN. We found no statistically significant difference between types of TN in respect to gender or age. Number of previous procedures was higher in type 2 TN (6 out of 11 in type 2 TN and 6 out of 22 in type 1 TN), but it was not significant. The mean duration of symptoms prior PBC was in type 1 TN seven years and in type 2 TN eight years. Hypoesthesia after PBC was found in 13 (59%) cases of type 1 TN and six (55%) in type 2 TN. Duration of analgesia after PBC was in type 1 TN 15 ± 15.4 months and in type 2 TN 12 ± 7.6 months (*P* = 0.38). Analgesic consumption after 12 months was none or lower in type 1 TN in 17 cases (77%) and in type 2 TN in six cases (55%; *P* = 0.06).

### 3.2. Correlation between Parameters and Outcome

We analyzed correlation between clinical parameters before PBC and outcome of the procedure (duration of analgesia and use of analgesic 12 months after PBC). Outcomes were compared in dependence of gender, age of patients (60 years and younger versus 61 years and older), duration of symptoms (5 years and less versus 6 and more years), and previous surgical treatment. None of the parameters showed statistically significant correlation with outcomes. However, mean duration of analgesia was longer in females, younger patients and shorter duration of symptoms before PBC. According to previous surgical treatment mean duration of analgesia was almost the same with or without previous surgical treatment. Use of analgesic at 12 months was generally with all parameters lower than before PBC. Results are summarized in Table 1. Additionally, correlation between the length of balloon inflation and the duration of pain-free period was not statistically significant (*P* = 0.76).

### 3.3. Complaints and Complications

No major complications occurred in our group of patients. Non-ideal shape of the balloon was reported in 8 (24%) procedures. A mean duration of analgesia was longer in patients with ideal pear-shape of balloon at the time of the procedure (17.4 ± 13.7 months) compared to non-ideal shape (4.4 ± 4.0 months) (*P* = 0.01). In seven out of 33 procedures premature balloon rupture occurred, without any immediate consequences for the patients. Three patients developed cheek hematoma; this subsided with cold compression. In nineteen cases mild to moderate degree of transient ipsilateral facial sensory loss—numbness was noted.

## 4. Discussion

The patient's characteristics in our group of TN patients were comparable to other studies: female predominance, advanced age, and more commonly affected right side are commonly reported [9]. Procedures were performed by Mullan's technique with some modification as described in Section 2 [12]. During procedure it is important that, when inflated, the shape of balloon must be pear shaped. This shape has, according to Mullan and other researches, significant impact on the outcome [14–16]. Pear-shaped balloon correspond to the inner anatomy of the Meckel cave—the dural protrusion surrounding the trigeminal ganglion and the distal part of the trigeminal root [14]. This was also the case in our group of patients where better outcome was noted in those patients with ideal shape of the balloon. Another important predictor is also the duration of balloon compression. Al-Banyan et al. have found that long balloon inflation times of 20 min significantly improve the duration of pain-free period [17]. But on the other hand, longer compression times are associated with higher complication rates [15]. We can conclude that for satisfactory outcome during compression balloon should be pear-shaped and duration of compression need to be long enough to give us acceptable correlation between duration of pain-free period and side effects. Immediate pain relief after PBC is high. In our study it was similar to other studies, where immediate pain relief is reported in 83 to 100% of cases. Mullan and Lichtor report initial relief in all patients except in one [12]. Skirving and Dan reported in their large study with 531 PBC, initial pain relief in 521 PBC except in one and 9 procedure failures [18], 100% initial relief is reported by Natarajan [19].

Average duration of pain-free period with PBC is around 2 years, and recurrence rate for TN varies among studies. Mean pain-free period in our group of patients was within the range reported in other larger studies where mean duration of pain-free period ranges from 7.3 months to 30 months. Shorter duration is reported in studies with only recurrent TN [20, 21]. Reports on recurrence rate are dependent of duration of follow-up period. Unfortunately there is lack of long-term studies that could give us information about long-lasting outcome after PBC. Mullan and Lichtor predicted 20% recurrence rate over 5 years [12]. Two larger studies Baabor and Perez-Limonte with 230 PBC, reported 15% recurrence after 3 years [22], and Skirving and Dan reported 31.9% of recurrence in entire study lasting for 20 years [18]. Higher recurrence is reported by Kouzounias et al. with 50% recurrence at 21 months, and in both studies with recurrent TN (45.5% and 64%) [20, 21, 23]. 

Use of analgesic one year after PBC is lower than before. In our study majority of patients after one year needed no or less analgesic than before PBC. In studies where cases of repeated PBC in refractory TN were analyzed, the outcome as immediate pain relief and duration of analgesia was comparable with first-time procedures [20, 21, 24].

Some studies have shown longer duration of analgesia in type 1 TN [24, 25]; in others authors did not find differences between both types [26]. In our group of patients the outcome, according to duration of analgesia and postprocedure analgesic consumption, was better in type 1 TN, but not statistically significant. However, in our group of type 2 TN were more cases with previous procedures and longer duration of symptoms before PBC, which are two factors that are by some authors connected with worse outcome.

Currently there are no clinical parameters that could certainly predict which patients would have better outcome after PBC. In our study only some mild differences were shown. Age, gender of patient, affected trigeminal division, and side of TN, have shown in all studies no statistically significant impact on outcome after PBC [3, 15, 17]. On the other hand, shorter duration of symptoms before PBC (less than 5 years) is considered to be a favorable prognostic factor. Longer preprocedure symptoms duration is connected with higher risk of recurrence and complication rate [15, 19, 24]. If compression on trigeminal root and ganglion is excluded by MRI or patient is not willing to undergone MVD, PBC could be one of the first options. The history of previous treatment should not be obstacle for PBC and it should be considered in patients with recurrent TN [3, 13, 17, 23]. On the other hand, some studies have shown slightly higher complication rate in recurrent cases [15]. Postprocedure numbness is a predictor of longer duration of analgesia and less recurrence [3, 15]. Conclusions about predictive factors from our study are not reliable, due to retrospective nature of the study and small number of patients.

In PBC the incidences of complications is low and major complications are rare. In our study there were no major complication and no permanent morbidity, this corresponds to results of other studies. PBC can relieve pain in any of the three trigeminal divisions [12]. During procedure, when the foramen ovale is engaged, short-lasting bradycardia and the masseter and pterygoid muscles contraction are noted [15]. With additional use of local anesthetic we can lower the number of cardiovascular events [27]. One important issue is also premature balloon rupture which might affect the outcome of the procedure. The possible causes of this complication can include material fatigue, careless handling of the catheter before the introduction, or inflation with too much contrast medium. After procedure, hypoesthesia, is reported by almost all patients and it is usually mild to moderate and transient [13, 14, 19, 26, 28]. The goal of PBC is to injure the myelinated fibers that mediate the “trigger” for the lancinating pain of TN, and hypoesthesia is therefore the known consequence [3]. In some cases hypoesthesia is prolonged and permanent or even dysesthesias can occur [3, 13, 15, 19, 20, 29]. In severe and rare cases anaesthesia dolorosa has also been reported in the literature [20, 26]. So only light compression is safer in terms of dysesthesias, but will result in recurrences whereas a firm compression will eliminate recurrences but increase the risk of dysesthesias [12]. Having postoperative hypoesthesia is usually better tolerated in patients than recurrent pain [16]. One study suggest that with intraluminal monitoring of compression pressure and limiting the duration of compression it is possible to reduce the incidence of dysesthesias, severe numbness, and masseter weakness after PBC [28]. Other possible rare complication in PBC are masseter muscle weakness with mastication weakness, loss of corneal reflex, corneal keratitis, altered taste sensation, herpes perioralis eruption, hearing loss, transient sudden blindness, abducens weakness, transient trochlear nerve palsy, loss of olfactory sense, changes in lacrimation, AV fistula, subarachnoid hemorrhage, and aseptic meningitis [3, 9, 15]. Generally PBC bears little discomfort for patient and only short hospitalization (2-3 days) [12, 16, 29].

## 5. Conclusions

To sum up, when patients fail medical management, either due to intolerable side effects or loss of effect, early surgical options should be considered. There is no absolute guideline for selecting the best procedure for patients. Patient selection and procedure selection consider (1) the patient's medical condition, (2) previous procedures, and (3) the patient's willingness to accept the associated risks and benefits of each procedure.

Percutaneous techniques are recommended for elderly patients with higher operative risk factors. In general, PBC should be reserved for patients rejecting or not suitable for MVD. Contraindications for PBC are rare. Caution is needed in the case of the contrast agent allergy because rupture of the balloon catheter can occur. In case of recurrence after PBC, the patient may be treated with carbamazepine. It provides satisfactory relief at a tolerably low dose. If carbamazepine fails, PBC may be repeated. Advantages of PBC with comparison of other surgical options for treatment of TN are minimal invasiveness, high portion of pain-free patients after procedure, and low incidence of complications.

## Figures and Tables

**Table 1:** Patients' parameters and outcome of the procedure.

		Mean duration of analgesia (months)	Use of analgesic at 12 month	
			None or less	The same or more
Gender	Male (*n* = 8)	9 ± 7.7	5	3
	Female (*n* = 19)	16 ± 15.7	13	6
Age	60 and younger (*n* = 14)	16 ± 18.8	8	6
	61 and older (*n* = 19)	13 ± 7.5	15	4
Duration of symptoms	5 years and less (*n* = 17)	16 ± 16.4	14	3
	6 years and more (*n* = 16)	12 ± 9.1	9	7
Previous surgical treatment	Yes (*n* = 12)	15 ± 8.8	8	4
	No (*n* = 21)	14 ± 15.5	15	6
